# Measuring Resilience in Long-term Sick-listed Individuals: Validation of the Resilience Scale for Adults

**DOI:** 10.1007/s10926-023-10100-y

**Published:** 2023-03-27

**Authors:** Martin Inge Standal, Odin Hjemdal, Vegard Stolsmo Foldal, Lene Aasdahl, Roger Hagen, Egil A. Fors, Frederick Anyan

**Affiliations:** 1 Faculty of Medicine and Health Sciences, Department of Public Health and Nursing, Norwegian University of Science and Technology (NTNU), Trondheim, Norway; 2 NTNU Social Research, Trondheim, Norway; 3 Faculty of Social and Educational Sciences, Department of Psychology, Norwegian University of Science and Technology (NTNU), Trondheim, Norway; 4 Unicare Helsefort Rehabilitation Centre, Rissa, Norway; 5 Faculty of Social Sciences, Department of Psychology, University of Oslo, Oslo, Norway; 6 Modum Bad Research Institute, Vikersund, Norway

**Keywords:** Psychological resilience, Long-term sickness absence, Return to work, Measurement invariance, Validation

## Abstract

Return to work from long-term sick leave is influenced by personal and social factors, which can be measured by resilience, a construct that describe healthy adaptation against adversity. This study aimed to validate the validity and psychometric properties of the resilience scale for adults in a sample of long-term sick-listed individuals, and to investigate measurement invariance when compared with a university student sample. Confirmatory factor analysis was used on a sick-listed sample (*n* = 687) to identify the scale?s factor structure, and comparison with a university student sample (*n* = 241) was utilized to determine measurement invariance. Results show that a slightly modified factor structure, in accordance with previous research, achieved acceptable fit in the sick-listed sample, while comparisons with the student sample supported measurement invariance. This means that the study to a large degree support the factor structure of the resilience scale for adults in long-term sick-listed. Furthermore, the results indicate that the scale is similarly understood among long-term sick-listed as in a previously validated student sample. Thus, the resilience scale for adults can be a valid and reliable measure of protective factors in the long-term sickness absence and return to work context, and the subscale and total score can be interpreted similarly in long-term sick-listed as in other populations.

**Supplementary Information:**

The online version contains supplementary material available at 10.1007/s10926-023-10100-y.

## Background

Long-term sick leave is costly for society and is thought to be detrimental for the sick-listed individual [1]. Assisting sick-listed individuals back to work is a priority in several countries [2, 3]. In order to help individuals return to work (RTW), it is useful to know why some return while others do not, and why some return faster than others. Differences in health problems alone are not sufficient to explain differences in RTW rates [4]. Personal and social factors such as self-efficacy [5], perceptions and RTW expectations [6, 7], and a supportive social environment are also important for managing RTW [8]. Thus, RTW from long-term sick leave is influenced by the interaction between biological-, psychological-, and social factors [9, 10].

One construct that may be helpful to describe psychological and social factors involved in overcoming adversity is resilience. Resilience can be described as healthy adaptation despite adverse circumstances [11]. The research on resilience, spanning several decades [12, 13], has described a range of psychological and social factors that cluster around three domains: (1) the individual’s personal traits, skills, abilities or attitudes; (2) family resources, stability or cohesion that provide empathy and support, and (3) access to social or environmental resources that support a healthy response to adversity [14]. Given the role resilience has in healthy adaptation despite adversity, resilience could potentially also influence the propensity to remain at work despite ill health. Thus, the concept has also recently gained some attention in the RTW context [15]. There are indications that resilience increases the functioning of those struggling with pain [16, 17], and those with chronic illness [18]. However, research on resilience in the context of long-term sick leave or return to work is still lacking.

There are several scales that can be used to assess resilience [19]. One is the Resilience Scale for Adults (RSA), developed by Friborg, Hjemdal and colleagues [20, 21]. This scale is one of the few that validly covers all three domains referred to above [22] through subscales measuring *perception of self, planned future, social competence, structured style, family cohesion*, and *social resources*. The RSA is also one of the psychometrically better scales among those available [19], and the psychometric properties of the RSA have been validated in several countries [23–30]. Previous research has also shown construct validity, such as negative correlations with measures of loneliness [27], anxiety and depressive symptoms [25], and positive correlations with sense of coherence [25]. Evidence using the RSA also suggests that resilience could reduce the impact of stress on mental health problems [31]. In validation studies of the RSA gender differences have repeatably been identified in the subscales. Most notably with women scoring higher on social resources and men scoring higher on the subscale measuring self-perception [23–26, 28, 29, 31]. However, despite the robust psychometric properties, some discrepancies have also been found regarding the factor-item patterns. For instance, the initial factor structure of Jakobsen et al. [27] showed mediocre relative fit, which was helped by moving the *“becoming informed if a family member experiences a crisis”* item from the social resources subscale to the family cohesion subscale. Similarly, several others have also reported poorer factor loadings for this item [14, 24, 26].

To determine the usefulness of resilience in the long-term sick leave and RTW context, there is a need to understand whether the construct can be transferred to this new population. When using the same measurement scale across different populations, the meaning and function of the construct may differ [32]. Thus, validation and assessing measurement invariance, or the equivalence of a construct across groups, is argued to be an important first step [32, 33]. The most important types of measurement invariance when comparing samples on a scale are: (1) configural invariance (equal factor structure), (2) metric invariance (equal factor loadings), and (3) scalar invariance (equal intercepts). Configural invariance is the weakest requirement and testing for this simply examines if the same factor models may be assumed across samples. Support of configural invariance indicates that similar latent constructs have been measured in both groups. However, the factor loadings may still differ which testing for metric invariance will assess [34]. Support of metric invariance determines that factor loadings are equal across samples. This will indicate that a linear increase in the summarized raw score measures a comparable increase in the latent construct across samples [34]. This means that both samples will interpret the scale items similarly. Finally, testing for scalar invariance will assess whether the latent intercepts are equal [35]. Support for scalar invariance means that both samples use the same starting point for scaling their responses and will use the response categories comparably.

To further understand the construct of resilience, the aim of this study was to examine the validity and psychometric properties of the RSA in a sample of long-term sick-listed individuals. Furthermore, the study aimed to determine measurement invariance when compared with a student sample. Specifically, comparing samples we hypothesized that:

1) The six-factor structure of the RSA was expected to replicate across both groups (support for configural invariance), as well as observing comparable factor loadings in both groups (support for metric invariance). We did not expect full scalar (equal intercepts) as this is seldomly supported [34].

Furthermore, in the long-term sick-listed sample we hypothesized that:

2) In line with previous research, men were expected to report higher levels of perception of self while women were expected to report higher levels of social resources.

3) Significant negative correlations were expected between resilience total score, as well as the subscales, and symptom measures of anxiety and depression.

4) Finally, it was expected that the RSA would show incremental validity over and above symptoms of anxiety when predicting depressive symptoms.

## Method

This is a cross-sectional study utilizing data from two separate samples: A sample of Norwegian long-term sick-listed individuals, and a Norwegian student sample.

### Participants and Recruitment

#### Sample 1 – Norwegian Long-term Sick-listed Sample

Participants in the sick-listed sample were employed workers with any diagnoses aged 18–62 at eight weeks of current sick leave with a leave status of 50–100%. Exclusion criteria were pregnancy-related sick-leave or not having an employer (i.e., unemployed or self-employed). Participants were part of a cohort of sick listed workers included in a randomized controlled trial [36]. The study was approved by the Regional Committee for Medical and Health Research Ethics in South East Norway (No: 2016/2300). Written informed consent was obtained from all participants. Eligible participants living in Trondheim, Central Norway, were invited to participate in the study via the Norwegian Labour and Welfare Administration’s electronic communication site. All participants who accepted to participate in the trial were included in the present study (from August 2017 until October 2020). During this period 883 accepted invitation and received a web-based questionnaire by e-mail (15% of those invited). This questionnaire was answered by 688 (78%) of the included participants. One participant withdrew their data from the study leaving 687 participants for the present study. The mean age of the sample was 44.2 years (SD 10.0), and 64% were female. Diagnoses as categorized by the ICPC-2 [37] were split at about one third musculoskeletal (38%), one third psychological (30%), and one third for all other diagnoses (32%). Data in this sample were collected at baseline, prior to randomization in the trial.

#### Sample 2 – Norwegian University Student Sample

A second sample of university students (*n* = 241) participating in a separate study was included in the analyses of RSA measurement invariance. The project was approved by the Regional Committee for Medical and Health Research Ethics of Middle Norway (Reference: 2016/339). Participants were social sciences students at the Norwegian University of Science and Technology. Participation was voluntary and participants could withdraw their informed consent at any time, without consequences. The mean age of the sample was 25.4 years (*SD* 6.5). One hundred and forty-nine, 149 (61.8%) were females, 91 (37.8%) were males. One person did not report gender. At the time of data collection, 170 (70.5%) were studying and 69 (28.6%) were not. Two participants did not report their students’ status.

### Measurement Instruments

#### Resilience Scale for Adults

Resilience was assessed using the Resilience Scale for Adults [20, 21]. The scale consists of 33 questions within six subscales The subscales are: (1) *Perception of self*, which assesses the individual’s confidence in their ability to manage or cope with adverse life events. (2) *Planned future* assesses whether the individual has a positive outlook on their future, and whether they have a preference for making plans and creating goals that they believe can succeed. (3) *Social competence* assesses the individual’s ability to engage socially, feel at ease in social settings, and their flexibility in social interactions. (4) *Structured style* assesses the individual’s preference for establishing routines, planning ahead, and approaching tasks in an organized manner. (5) *Family cohesion* assesses shared family values, views of the future, family loyalty, and mutual appreciation. (6) *Social resources* assesses to what degree the individual has resources outside the family that may provide encouragement and assistance if need be [25]. The items are scored on a scale from 1 (low) to 7 (high), and the RSA is usually scored with a mean score of the 33 items to estimate psychological resilience.

#### Anxiety and Depression Scales

Anxiety was assessed using the Generalized Anxiety Disorder-7 (GAD-7) questionnaire [38], and depression with the Patient Health Questionnaire-9 (PHQ-9) [39]. GAD-7 and PHQ-9 uses four-point categorical scales to assess whether individuals have experienced anxiety and depressive problems in the previous two weeks. The categories are “not at all”, “some days”, “more than half of the days”, and “almost every day”. Higher scores indicate more anxiety and depressive symptoms.

### Analysis

Basic correlation, group mean difference tests and regression analyses were performed in SPSS version 28 (IBM Corp, 2021). All other analyses were performed in Mplus version 8.7 (Muthén & Muthén, 1998–2021) using robust full-information maximum likelihood. As a first step, a well-fitting Confirmatory Factor Analysis (CFA) model was established in single-group analysis across both groups. Measurement invariance was then conducted. Configural invariance was tested first, which also represented the baseline model for the subsequent and more restrictive models. Metric invariance was tested by constraining all factor loadings as equal across groups. Next, item intercepts were constrained equal across the groups to test scalar invariance. Since full scalar invariance is seldomly supported, non-invariant intercepts with high modification indices were identified, thus improving model fit significantly if freed. As the increasingly restrictive models estimate the same parameters as the unconstrained models, change in model fit was examined. In accordance with Chen [34], we examined a change of ≥ − 0.010 in CFI, and ≥ 0.015 in RMSEA or a change of ≥ 0.030 in SRMR as indicating non-invariance when testing metric invariance. For testing scalar invariance, we used the same changes in values for CFI and RMSEA, supplemented by a change of ≥ 0.010 in SRMR as indicating non-invariance. Incremental validity of the RSA was tested in two ways; (i) by using the popular stepwise regression approach to determine the additional contribution of the RSA total score and the subscales as separate predictors of depressive symptoms over and above symptoms of anxiety, (ii) by using the less common but efficient SEM approach, which accounts for measurement error unlike the regression approach. Using the regression approach can produce misleading results [40]. Established and widely accepted recommendations for testing incremental validity in SEM (e.g., Wang and Eastwick [40]) were followed.

## Results

### Mean Differences in RSA Scores Across Samples and Gender

Table 1 presents the means, standard deviations and reliability estimates of the RSA with subscales.

**Table 1:** Table of Means, Standard deviations, Test Score Reliability and Pearson’s Correlation between RSA subscales

	Variable	Sick-listed *n* = 687		Students *n* = 241			Reliability Sick-listed	Correlation coefficients						
		*Mean*	*SD*	*Mean*	*SD*	*g*	*α*	1	2	3	4	5	6	7
1	RSA Total	5.09	0.93	5.04	0.86	0.07	0.86		0.82	0.78	0.76	0.75	0.79	0.58
2	Perception of self	4.67	1.37	4.48	1.28	0.14*	0.86	0.73		0.71	0.56	0.44	0.48	0.43
3	Planned future	4.43	1.54	4.95	1.47	− 0.35***	0.84	0.79	0.68		0.50	0.39	0.48	0.44
4	Social competence	4.93	1.19	4.94	1.17	0.00	0.79	0.69	0.37	0.42		0.41	0.55	0.33
5	Family cohesion	5.43	1.17	5.20	1.18	0.19**	0.86	0.70	0.29	0.36	0.36		0.66	0.33
6	Social resources	5.86	1.09	5.74	1.06	0.12	0.86	0.79	0.43	0.52	0.55	0.58		0.34
7	Structured style	4.93	1.05	4.76	1.26	0.15*	0.50	0.53	0.31	0.48	0.19	0.25	0.24	

*Note*: ***p < .001; **p < .01; *p < .05, *g* = Hedge’s g (effect size), *α* = Cronbach’s alpha, Correlation coefficients between the RSA subscales are shown above the diagonal for the sick-listed sample and below the diagonal for the student sample. Correlations are significant at *p* < .01

In the sick-listed sample, women compared to men reported slightly higher levels of resilience total (Mean: *M* = 5.12 vs. *M* = 5.06, *t* = 0.78, *p* = .217), social competence (*M* = 4.98 vs. *M* = 4.85, *t* = 1.26, *p* = .103), family cohesion (*M* = 5.40 vs. *M* = 5.34, *t* = 0.57, *p* = .283), social resources (*M* = 5.94 vs. *M* = 5.69, *t* = 2.94, *p* < .01), and structured style (*M* = 4.98 vs. *M* = 4.82, *t* = 1.85, *p* < .05). Men compared to women reported higher levels of perception of self (*M* = 4.81 vs. *M* = 4.60, *t* = 1.82, *p* < .05) and planned future (*M* = 4.52 vs. *M* = 4.38, *t* = 1.15, *p* = .125). Thus, significant gender differences were found for social resources and perception of self in support of hypothesis (2).

#### Configural Invariance

The original six-factor structure did not reach acceptable fit. The slightly modified six-factor structure by Jakobsen et al. [27], which moved item 23 *(becoming informed if a family member experiences a crisis)* from the *social resources* factor to the *family cohesion* factor, and allowing two correlated error terms between items 15 *(ease of finding new friendships)* and 21 *(good at coming in contact with others)*, and 12 *(seldom planning before doing something)* and 24 *(routines are lacking in my daily life)* showed an improvement, reaching acceptable fit in the sick-listed (*χ*^*2*^ = 1155.403, *df* = 478, *p* < .001; SRMR = 0.046; RMSEA = 0.047 [90% CI = 0.043, 0.050]; CFI = 0.912; TLI = 0.903) and the student samples (*χ*^*2*^ = 792.956, *df* = 478, *p* < .001; SRMR = 0.063; RMSEA = 0.052 [90% CI = 0.046, 0.059]; CFI = 0.900; TLI = 0.889) (M1a and M1b, Table 3). Inspection of modification indices in both samples also supported moving item 23 onto the family cohesion factor. Configural invariance (M2) was adequate as the equivalent six-factor model in both samples where identical factor structure had acceptable fit in terms of the RMSEA and CFI indices. See Table 2 for standardized factor loadings and Table 3 for evaluations of measurement invariance.

**Table 2:** Standardized Factor Loadings in both samples

Sick-listed (*n* = 687)								Students (*n* = 241)					
*Items*	1	2	3	4	5	6		1	2	3	4	5	6
PS 1	0.72							0.68					
PS 2	0.74							0.67					
PS 3	0.70							0.66					
PS 4	0.75							0.80					
PS 5	0.71							0.70					
PS 6	0.63							0.58					
PF 1		0.75							0.77				
PF 2		0.76							0.81				
PF 3		0.75							0.82				
PF 4		0.73							0.69				
SC 1			0.55							0.48			
SC 2			0.41							0.49			
SC 3			0.71							0.70			
SC 4			0.74							0.82			
SC 5			0.57							0.54			
SC 6			0.68							0.63			
FC 1				0.70							0.63		
FC 2				0.73							0.78		
FC 3				0.76							0.75		
FC 4				0.72							0.65		
FC 5				0.61							0.61		
FC 6				0.65							0.80		
FC 7				0.61							0.62		
SR 1					0.76							0.73	
SR 2					0.79							0.81	
SR 3					0.58							0.58	
SR 4					0.84							0.89	
SR 5					0.72							0.76	
SR 6					0.67							0.71	
SS 1						0.37							0.35
SS 2						0.08							0.28
SS 3						0.75							0.83
SS 4						0.52							0.74

*Note*: PS = Perception of self, PF = Planned future, SC = Social competence, FC = Family cohesion, SR = Social resources, SS = Structured style. Adjusted model with one item moved from SR to FC.

**Table 3:** Evaluations of measurement invariance between Sick-listed (n = 687) and Students (n = 241) samples

Model	Type of test	Compared with	*χ* ^*2*^	*df*	*RMSEA*	*CFI*	*TLI*	*SRMR*	*Δdf*	*ΔCFI*	*ΔRMSEA*	*ΔSRMR*	
M1a	Sick-listed		1155.40	478	0.047 [0.043, 0.050]	0.912	0.903	0.046					
M1b	Students		792.96	478	0.052 [0.046, 0.059]	0.900	0.889	0.063					
M2	Configural inv.		1962.53	956	0.049 [0.045, 0.052]	0.909	0.899	0.051					
M3	Metric inv.	M2	2002.70	983	0.048 [0.045, 0.051]	0.907	0.901	0.056	27	− 0.002	− 0.001	0.005	
M4	Scalar inv.	M3	2250.89	1010	0.052 [0.050, 0.055]	0.887	0.882	0.061	27	− 0.020	0.004	0.005	
M4a	Partial scalar inv.	M3	2096.16	1004	0.049 [0.046, 0.052]	0.901	0.896	0.057	21	− 0.006	0.001	0.001	

*Note: χ*^*2*^  *=* chi-square statistic, *df =* degrees of freedom, *RMSEA =* Root Mean Square Error of Approximation, *CFI =* Comparative Fit Index, *TLI =* Tucker Lewis Index, *SRMR =* Standardized Root Mean Square Error of Approximation, *Δ =* Change in statistical values. Inv. = invariance

#### Metric Invariance

The baseline model (M2) was compared to a model constraining the factor loadings equally across both groups (M3), thus testing the important assumption of metric invariance. The worsening of fit was trivial as indicated by the change in CFI and RMSEA (*Δ*CFI = − 0.002; *Δ*RMSEA = − 0.001); hence, both models were equivalent and supported metric invariance.

#### Scalar Invariance (Partial)

The fit of model M4 (equal item intercepts) was significantly worse than model M3 (allowing different intercepts) as expected; hence, not supporting strong invariance. The worsening in fit was substantial in *Δ*CFI = − 0.020, although minor in *Δ*RMSEA = 0.004 and *Δ*SRMR = 0.005. Lack of invariance in the latent intercepts involved six items, one item belonging to each of the *perceptions of self, planned future* and the *structured style* factors, and three items belonging to the *family cohesion* factor. Hence, partial scalar invariance was supported (M4a). These item intercepts were thus freely estimated in the following analyses.

#### Concurrent and Incremental Validity of the RSA in the Sick-listed Sample

As expected, the RSA total score correlated significantly negatively with measures of generalized anxiety symptoms (*r* = − .58, *p* < .01) and depressive symptoms (*r* = − .65, *p* < .01). The subscales of the RSA also correlated negatively with measures of generalized anxiety symptoms (ranging from − 0.29 to − 0.63) and depressive symptoms (ranging from − 0.35 to − 0.65). Incremental validity using the regression approach was assessed by identifying whether the increment in certainty of the prediction (*ΔR*^*2*^) was significant when resilience total score or a subscale was included in the model. In step 1, symptoms of anxiety (Standardized: *β* = 0.78, *SE* = 0.03, *t* = 31.35, *p* < .001) were significantly positively associated with depressive symptoms (*R*^*2*^ = 61%). Resilience total score in step 2 was associated with depressive symptoms (*β* = − 0.30, *SE* = 0.18, *t* = -10.50, *p* < .001), accounting for additional variance (*ΔR*^*2*^ = 5.8%). Results from substituting separate subscales in step 2 were as follows: *Perception of self* (*β* = − 0.25, *SE* = 0.13, *t* = -8.09, *p* < .001; *ΔR*^*2*^ = 3.7%), *Planned future* (*β* = − 0.28, *SE* = 0.11, *t* = -10.07, *p* < .001; *ΔR*^*2*^ = 5.4%), *Social competence* (*β* = − 0.15, *SE* = 0.12, *t* = -8.14, *p* < .001; *ΔR*^*2*^ = 2.0%), *Family cohesion* (*β* = − 0.17, *SE* = 0.13, *t* = -6.64, *p* < .001; *ΔR*^*2*^ = 2.5%), *Social resources* (*β* = − 0.18, *SE* = 0.14, *t* = -6.97, *p* < .001; *ΔR*^*2*^ = 2.8%) and *Structured style* (*β* = − 0.14, *SE* = 0.14, *t* = -5.29, *p* < .001; *ΔR*^*2*^ = 1.7%).

A minimum requirement in the SEM approach to testing incremental validity is to establish empirical evidence for construct separability between resilience and the other covariate (anxiety symptoms) in a CFA that compares a unifactorial model (resilience and anxiety symptoms are not separate constructs) to a two-factor model (resilience and anxiety symptoms are separate constructs). The unifactorial model was a poor fit (*χ*^*2*^ = 854.495, *df* = 65, *p* < .001; SRMR = 0.09; RMSEA = 0.14 [90% CI = 0.128, 0.144]; CFI = 0.78; TLI = 0.74) while the two-factor model showed an improvement (*χ*^*2*^ = 251.792, *df* = 63, *p* < .001; SRMR = 0.04; RMSEA = 0.07 [90% CI = 0.059, 0.077]; CFI = 0.95; TLI = 0.94). The factor correlation was significant (*r* = − .73, *p* < .001), providing strong evidence that resilience was a separate construct from anxiety symptoms. Incremental validity in the SEM approach (contained in the Supplementary material, Figure S1 to S7) was evidenced by significant path coefficient from resilience total score and subscales to depressive symptoms over and above symptoms of anxiety. Path coefficients were as follows: resilience total score (*β* = − 0.36, *p* < .001), perception of self (*β* = − 0.28, *p* < .001), planned future (*β* = − 0.31, *p* < .001), social competence (*β* = − 0.15, *p* < .001), family cohesion (*β* = − 0.17, *p* < .001), social resources (*β* = − 0.18, *p* < .001), and structured style (*β* = 0.17, *p* < .001).

## Discussion

The main aim of the current study was to examine the psychometric properties of the RSA in a sample of long-term sick-listed individuals. To a large degree the results support the validity of the RSA in the sick-listed sample and indicate that the questionnaire is similarly understood by long-term sick-listed individuals as in other populations.

The factor structure of the RSA has been tested in several previous studies and shows evidence of being a psychometrically valid questionnaire for assessing protective factors of resilience among adults [23–30]. The present study largely found support for the factor structure. However, using modification indices as a guide and moving the item assessing *“being informed if a family experiences a crisis”* from social resources to family cohesion was required to improve model fit to acceptable levels. This builds upon previous evidence that this item may for some samples fit better for describing family cohesion than social resources [27]. Following the trend demonstrated in previous validation studies [28, 29], the present study also revealed low internal consistency on the *structured style* subscale, indicating that all the items in this subscale may not capture the same construct adequately. Two correlated error terms between items 15 *(ease of finding new friendships)* and 21 *(good at coming in contact with others)* measuring S*ocial Competence*, and items 12 *(seldom planning before doing something)* and 24 *(routines are lacking in my daily life)* measuring *Structure Style* were freely estimated. While this is sensible due to the overlap in item content and semantic meaning of the subscales the items belong, we concede that this adjusted factor structure of the RSA may not generalize beyond our samples as this procedure often leads to models that do not cross-validate well.

The present study also showed configural invariance when compared with a student sample, meaning that the factor structure is comparable across samples. Furthermore, metric invariance was also supported, which indicates that a value change in the RSA score for a long-term sick-listed individual, can be compared with a similar value change in other samples. Scalar invariance means that long-term sick-listed individuals has the same starting point on the RSA as the other sample. Full scalar invariance requires that all item intercepts are equal across groups and is often not found [32, 34]. Scalar invariance was also not supported in the present study. This means that item means for the factors were not equivalent across these samples [32]. In the present study, only six out of 33 items failed to show scalar invariance which is quite good. However, the overall model showed substantially worse fit and thus scalar invariance was only partially supported. This could indicate that the context of being sick-listed creates different interpretations of question wordings than in a student sample. However, the most notable difference between the samples were sick-listed individuals reporting lower means for planned future than the student sample. This could also be due to uncertainty about the future for the long-term sick-listed [41], which possibly creates difficulties in planning ahead. Similar results have also been found when comparing clinical and nonclinical samples [28]. As scalar invariance was not being fully supported, direct comparisons of mean subscale scores between long-term sick-listed samples and other samples may be biased [32].

Expected gender differences in the RSA subscales were also found among long-term sick-listed individuals. Women reported higher levels of social resources and structured style, while men reported higher levels on perception of self. The differences in social resources and perception of self are often reported [23–26, 28, 29, 31], and the differences in structured style have also been found previously [24, 25, 29]. These results mean that future studies using the RSA should be mindful of these differences and take them into account in their analysis (e.g., by reporting the groups separately, or adjusting for gender).

The results also indicated that scores of resilience and all subscales were negatively correlated with anxiety and depression in the sick-listed sample, in support of our hypothesis. Such a correlation was expected as resilience is supposed to be a measure of protective resources associated with good adaptation against mental health issues [42]. This result provides further evidence of the concurrent validity of the RSA, also among long-term sick-listed individuals. Furthermore, the results also demonstrate incremental validity of the RSA beyond anxiety when predicting depressive symptoms. This indicates that the RSA measures something else than just the absence of mental health issues, i.e., the construct is not on the same scale, which is also aligned with previous findings [43]. Thus, the results show the discriminant validity of the RSA when compared to measures of mental health symptoms. This means that resilience can provide information on anxiety and depressive symptoms beyond being a correlated measure of anxiety and depression.

### Implications for Practice

The findings in the present study supports the validity of a slightly adjusted RSA to measure the protective factors of resilience in the current sample of long-term sick listed individuals. The use of the RSA and its subscales may provide important information on factors that have been shown to be important predictors of RTW, such as self-efficacy, social support, structure, and planning [5, 44, 45]. Gender differences in resilience resources, and early identification of those scoring high and low on such factors may be useful to determine appropriate and targeted interventions based on which resources (i.e., subscales) are lacking. However, this is an avenue that requires more research before implementing in practice.

### Strengths and Limitations

One strength of the current study is the relatively large sample size of the long-term sick listed sample. Furthermore, the study utilized two separate samples to assess measurement invariance, something that has been lacking in validation research [33].

However, the study does have some limitations. First, both samples might be highly selected. The student sample utilized convenience sampling, and while the long-term sick-listed sample invited all individuals that were eligible based on inclusion and exclusion criteria, recruitment achieved only a 15% participation rate. Nonetheless, demonstrating measurement invariance on these two different samples highlights that they understand the questionnaire similarly and does increase confidence in the validity of the RSA for use in RTW research, regardless of the limitations of each sample separately. As this is a cross-sectional study, other limitations are the inability to assess whether the RSA can predict actual RTW, and lack of possibilities to investigate test-retest validity. A longitudinal study with RTW outcome data should be performed to assess the predictive validity of the RSA on RTW. Furthermore, investigating the test-retest stability is required to determine whether the RSA is scored differently over time, for example depending on length of sick leave.

## Conclusion

The results from comparisons between a student sample and a sample of long-term sick-listed largely supports the factor structure of the RSA in long-term sick-listed individuals. There were only slight deviations from the expected factor structure, and by moving one item from the social resources subscale to the family cohesion subscale helped improve model fit to acceptable levels. Comparison between the samples also to a large degree demonstrated measurement invariance, which indicate that the RSA questionnaire is similarly understood among long-term sick-listed individuals in Norway as in a previously validated student sample. Thus, the results suggest that the RSA can be used to assess resilience among long-term sick-listed individuals and that the subscales and total scale score can be interpreted similarly to other populations.

### Electronic Supplementary Material

Below is the link to the electronic supplementary material.

Supplementary Material 1

## Data Availability

The datasets generated and analysed during the current study are not publicly available due to protecting the anonymity of participants.
