# Supplementary material for: Measuring Resilience in Long-term Sick-listed Individuals: Validation of the Resilience Scale for Adults
Source: J Occup Rehabil. 2023 Mar 27;33(4):713–22. doi: 10.1007/s10926-023-10100-y (PMC10684425; doi:10.1007/s10926-023-10100-y)

**Supplementary material**

**Figure S1**: Resilience total score path model


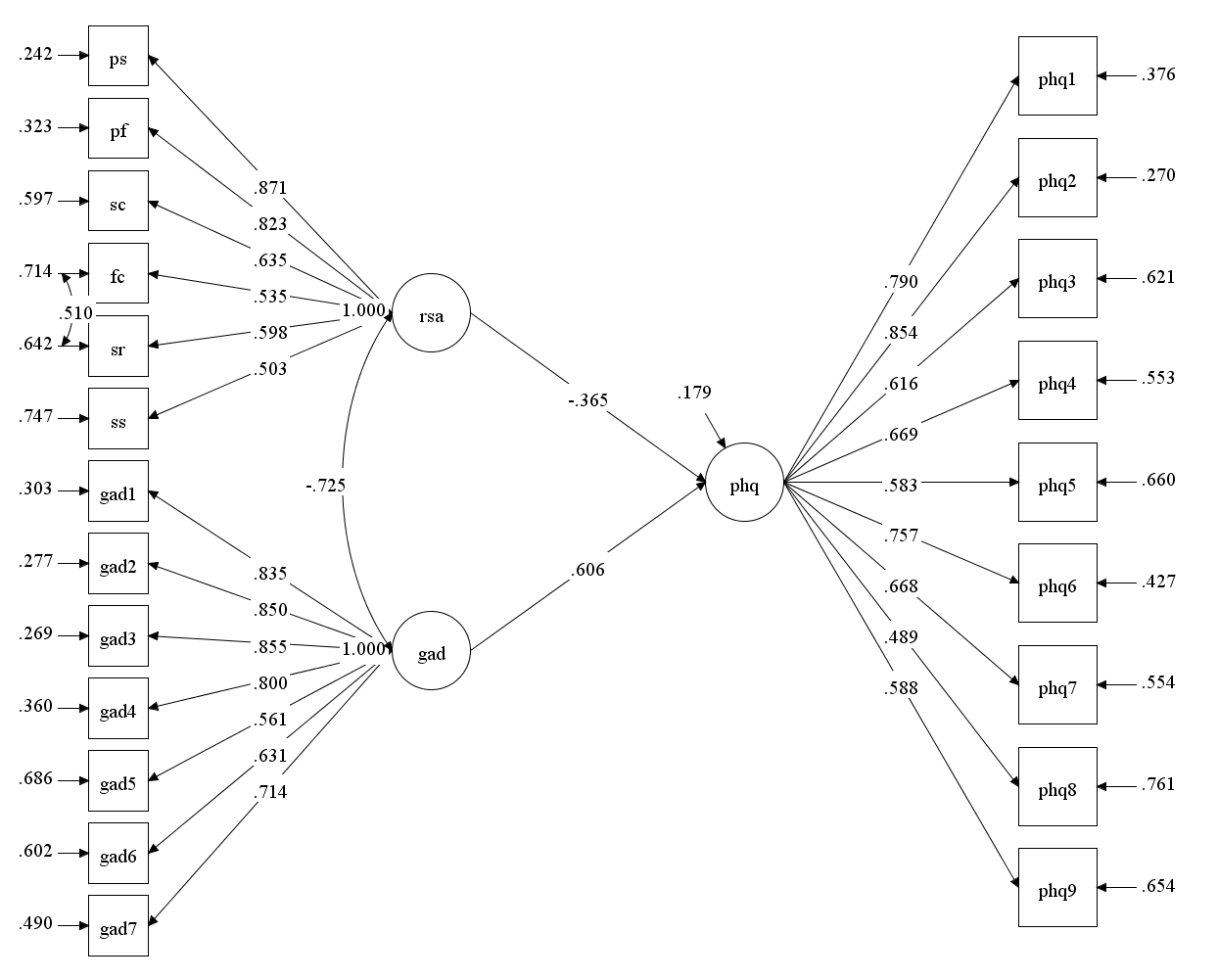


We explored a model with resilience total score where the subscales were used as factor indicators constructed through parcelling. Parcelling help to reduce method effects associated with individual items and random errors while increasing reliability of the structural model; hence parcelling is considered acceptable when conducting latent variable SEM with multiple indicators (Little et al., 2002; Wang & Wang, 2019].

Little TD, Cunningham WA, Shahar G, Widaman KF. To parcel or not to parcel: Exploring the question, weighing the merits. Struct Equ Model. 2002. 9(2): p. 151-173. Doi: <https://doi.org/10.1207/S15328007SEM0902_1>

Wang, J. and X. Wang. Structural equation modeling: Applications using Mplus. 2019, UK: John Wiley & Sons.

**Figure S2**: Perception of self (PS) path model


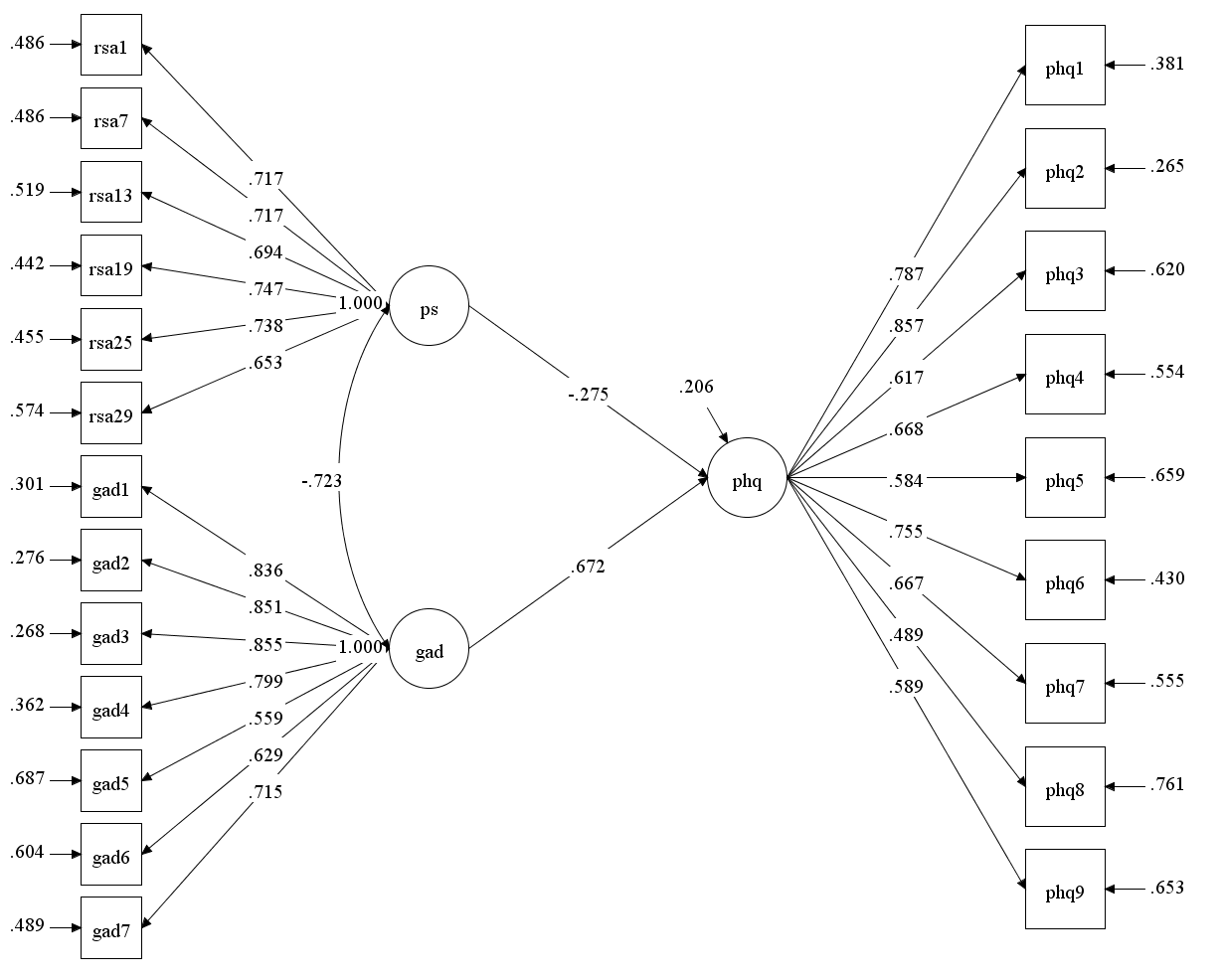


**Figure S3**: Planned future (PF) path model


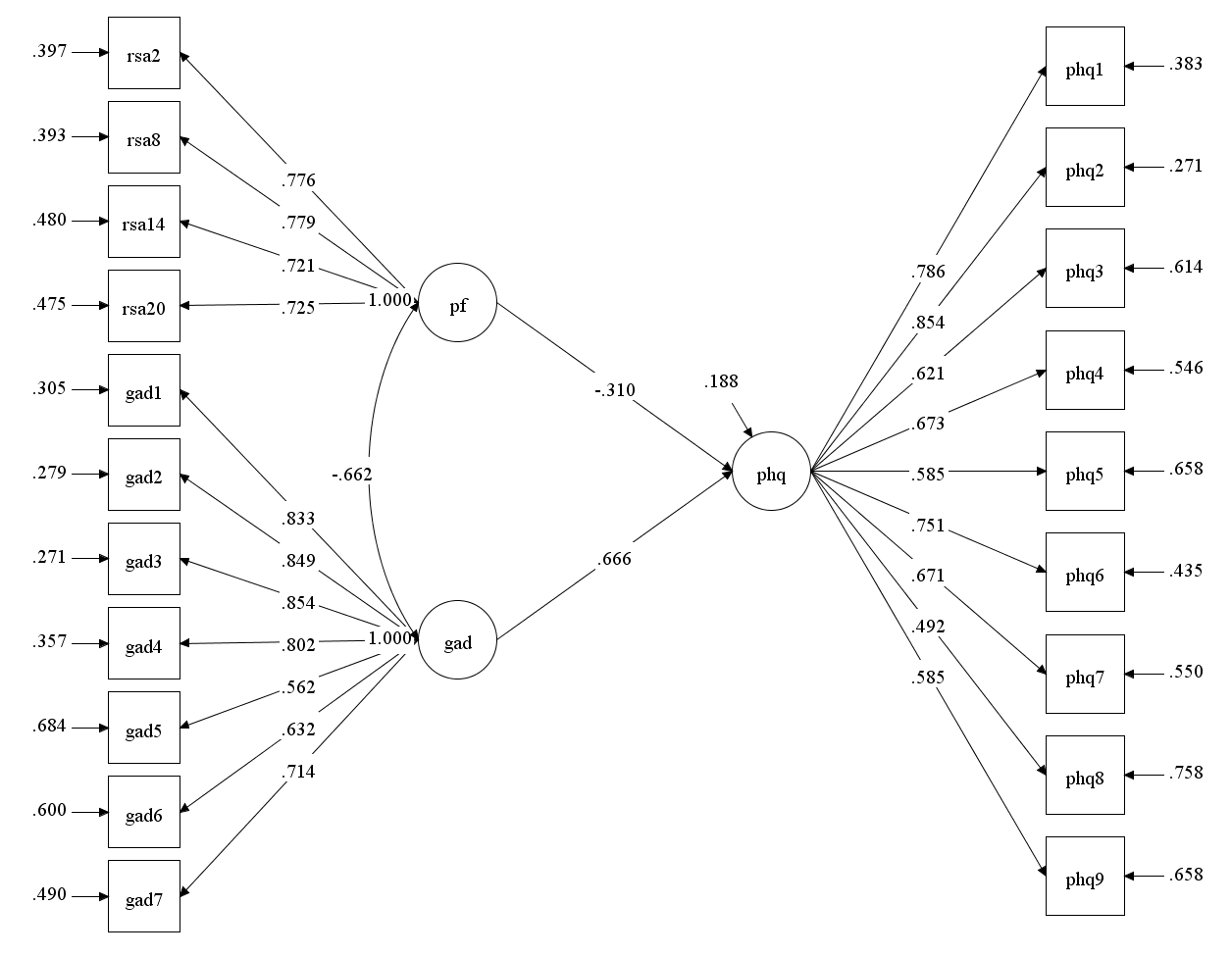


**Figure S4**: Social competence (PC) path model


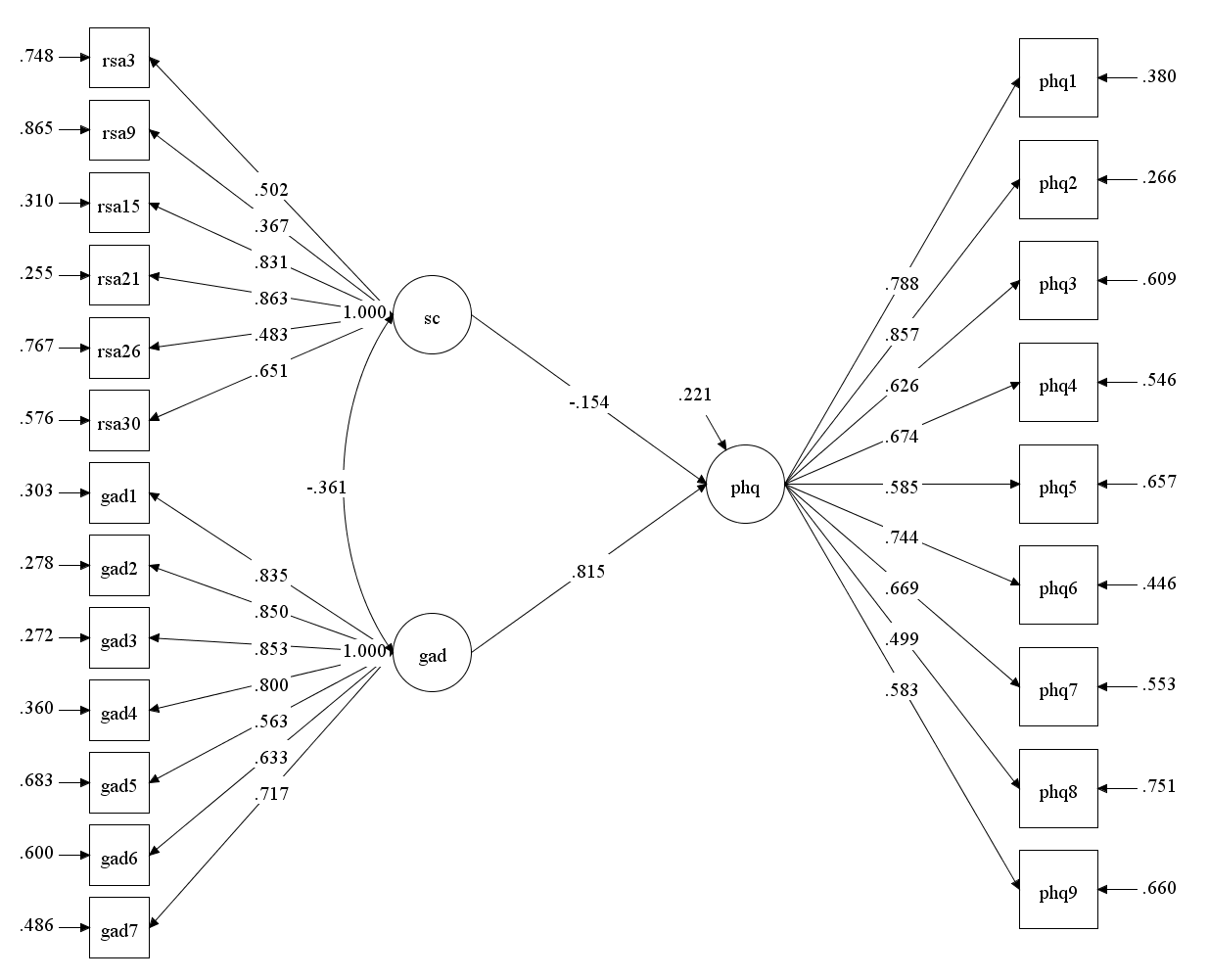


**Figure S5**: Family cohesion (FC) path model


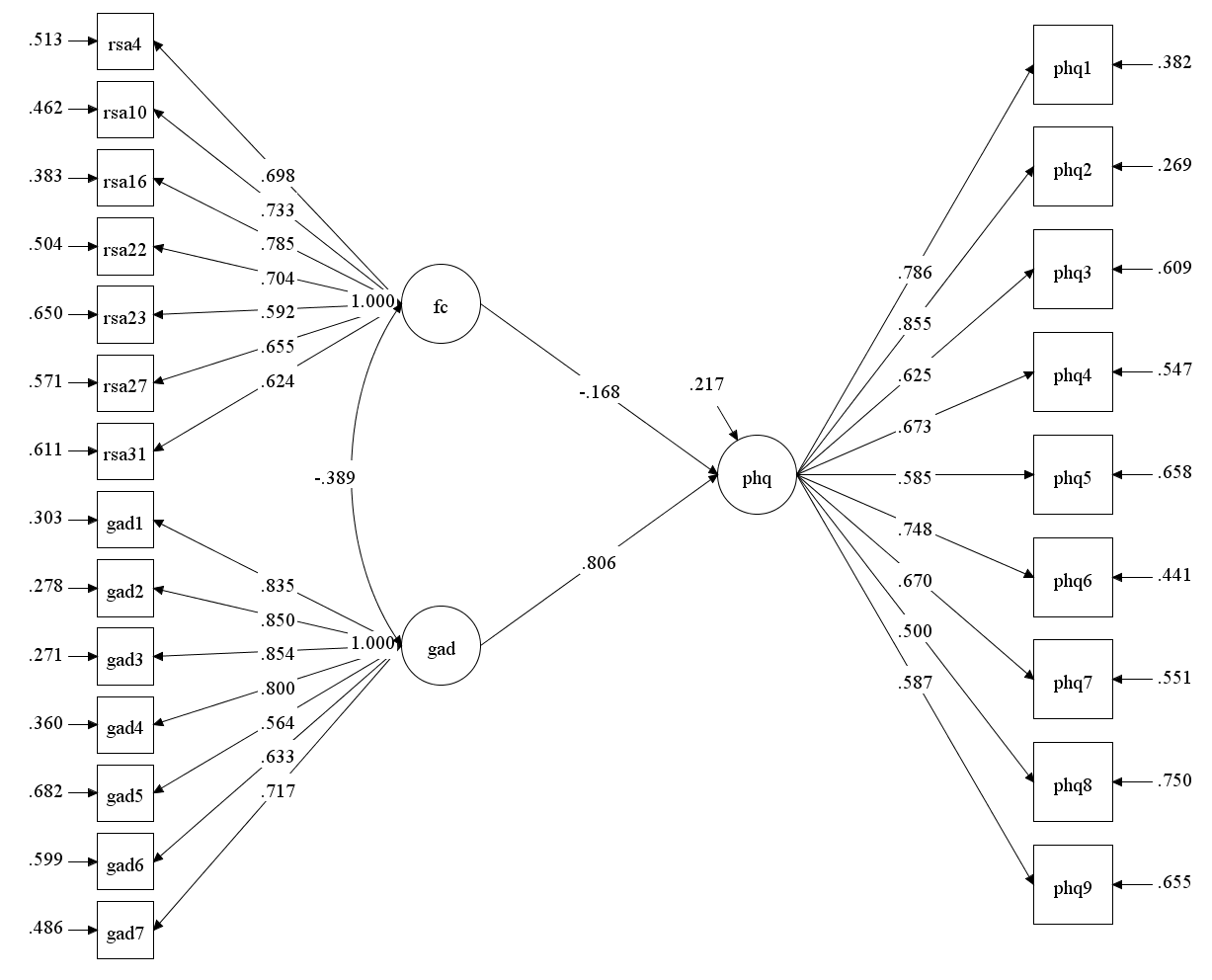


**Figure S6**: Social resources (SR) path model


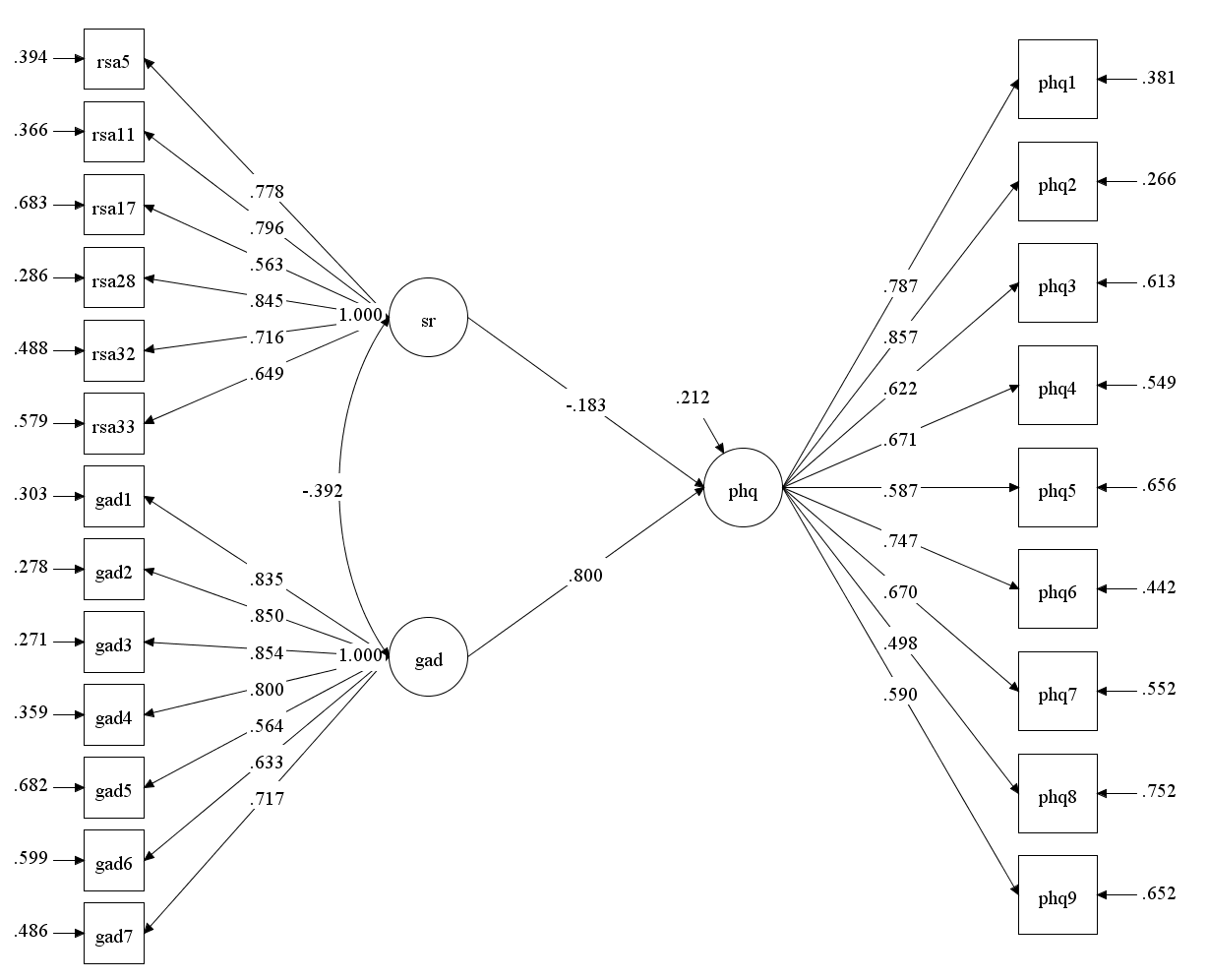


**Figure S7**: Structured style (SS) path model


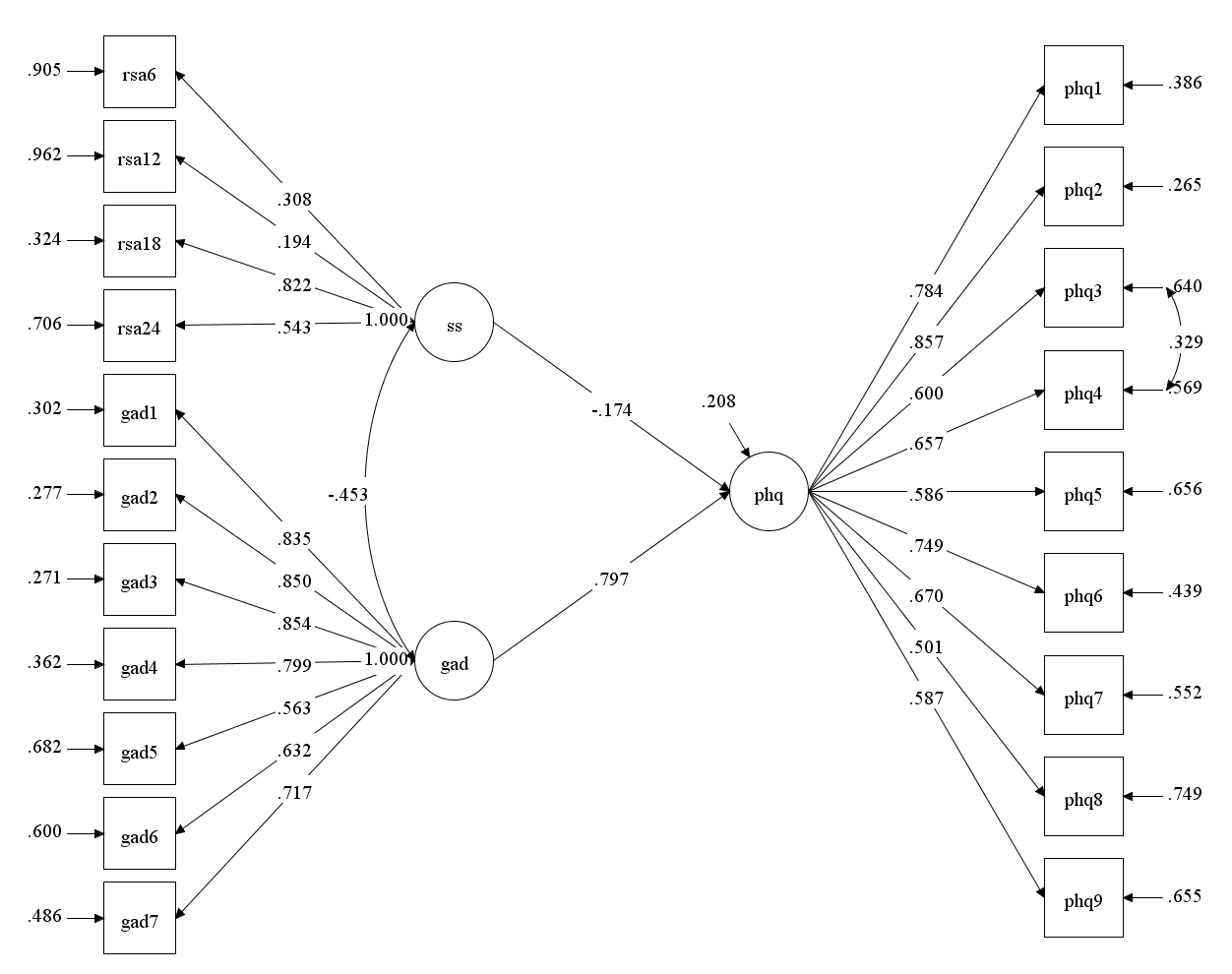

Supplement: Supplementary file 1 — Supplementary Material 1 [file 10926_2023_10100_MOESM1_ESM.docx]
